# Development of an Antibody Delivery Method for Cancer Treatment by Combining Ultrasound with Therapeutic Antibody-Modified Nanobubbles Using Fc-Binding Polypeptide

**DOI:** 10.3390/pharmaceutics15010130

**Published:** 2022-12-30

**Authors:** Yusuke Yano, Nobuhito Hamano, Kenshin Haruta, Tomomi Kobayashi, Masahiro Sato, Yamato Kikkawa, Yoko Endo-Takahashi, Rui Tada, Ryo Suzuki, Kazuo Maruyama, Motoyoshi Nomizu, Yoichi Negishi

**Affiliations:** 1 Department of Drug Delivery and Molecular Biopharmaceutics, School of Pharmacy, Tokyo University of Pharmacy and Life Sciences, 1432-1 Horinouchi, Hachioji, Tokyo 192-0392, Japan; 2 Department of Clinical Biochemistry, School of Pharmacy, Tokyo University of Pharmacy and Life Sciences, Tokyo 192-0392, Japan; 3 Laboratory of Drug and Gene Delivery Research, Faculty of Pharma-Sciences, Teikyo University, 2-11-1 Kaga, Itabashi-ku, Tokyo 173-8605, Japan; 4 Laboratory of Ultrasound Theranostics, Faculty of Pharma-Sciences, Teikyo University, 2-11-1 Kaga, Itabashi-ku, Tokyo 173-8605, Japan

**Keywords:** antibody delivery, ultrasound, nanobubbles, Fc-binding polypeptide, cancer

## Abstract

A key challenge in treating solid tumors is that the tumor microenvironment often inhibits the penetration of therapeutic antibodies into the tumor, leading to reduced therapeutic efficiency. It has been reported that the combination of ultrasound-responsive micro/nanobubble and therapeutic ultrasound (TUS) enhances the tissue permeability and increases the efficiency of delivery of macromolecular drugs to target tissues. In this study, to facilitate efficient therapeutic antibody delivery to tumors using this combination system, we developed therapeutic antibody-modified nanobubble (NBs) using an Fc-binding polypeptide that can quickly load antibodies to nanocarriers; since the polypeptide was derived from Protein G. TUS exposure to this Herceptin^®^-modified NBs (Her-NBs) was followed by evaluation of the antibody’s own ADCC activity, resulting the retained activity. Moreover, the utility of combining therapeutic antibody-modified NBs and TUS exposure as an antibody delivery system for cancer therapy was assessed in vivo. The Her-NBs + TUS group had a higher inhibitory effect than the Herceptin and Her-NBs groups. Overall, these results suggest that the combination of therapeutic antibody-modified NBs and TUS exposure can enable efficient antibody drug delivery to tumors, while retaining the original antibody activity. Hence, this system has the potential to maximize the therapeutic effects in antibody therapy for solid cancers.

## 1. Introduction

More than 100 antibody drugs have been approved worldwide for treating various diseases (e.g., cancer, infectious diseases, and chronic inflammatory diseases), and nearly half of these have been used in cancer therapy [1]. In addition, immune checkpoint-inhibiting antibodies and antibody–drug conjugates have ushered in remarkable progress in cancer treatment recently [2,3]. Thus, antibodies are central therapeutic agents for the treatment of cancer. The most distinctive feature of therapeutic antibodies is their high efficacy and minimal off-target toxicity. Therapeutic antibodies bind directly to tumor antigens on the cell membrane and exert strong anti-cancer effects through multiple mechanisms, such as inhibition of signaling pathways, antibody-dependent cellular cytotoxicity (ADCC), and complement-dependent cytotoxicity (CDC). ADCC is invoked when immune cells with Fc receptors recognize the Fc region of antibodies bound to cancer cells and release cytotoxic granules such as perforin and granzyme. CDC is initiated by C1q, the initiating component of the classical complement pathway, which reacts to the Fc region of antibodies bound to cancer cells [4]. However, the efficacy of therapeutic antibodies has been limited in solid tumors due to the poor distribution of antibodies (<0.1% of the injected dose/g of tumor) within tumor tissues [5,6]. This limitation is intricately related to the tumor microenvironment (TME), which has several physiological barriers, such as stromal barriers [7,8]. Stromal tissues are mainly composed of the extracellular matrix (ECM), such as collagen and hyaluronic acid, and specialized connective tissue cells, including fibroblasts, which promote passive diffusion from tumor tissues. To overcome physiological barriers in TME, antibody therapy in combination with ECM-manipulating agents (e.g., collagenase and hyaluronidase) has been explored in preclinical studies; for chemotherapy, such combination has also been explored in clinical studies [9,10,11,12,13,14].

**Figure 1:**
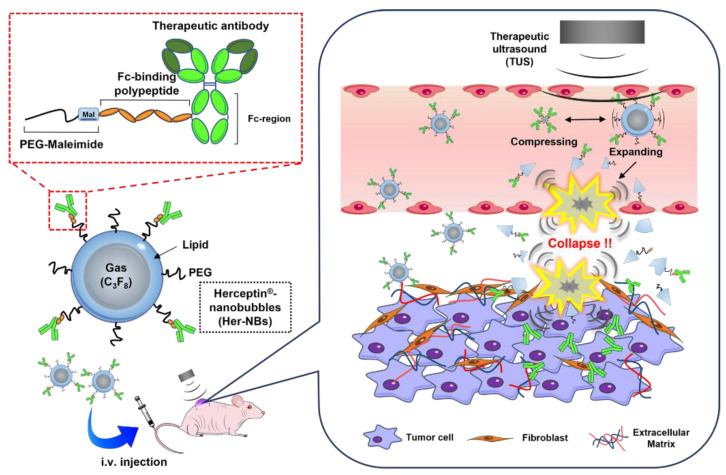
Schematic drawing of the structure of therapeutic antibody-modified nanobubbles (NBs) and the in vivo mechanism of action for the combination of therapeutic antibody-modified NBs and therapeutic ultrasound (TUS) exposure. Therapeutic antibody-modified NBs, which are loaded with Fc-binding polypeptide, expand to a much larger size upon TUS exposure and then collapse. The formation of jets caused by the collapse of NBs perforates surrounding blood vessels and membranes, enhancing the therapeutic antibody accumulation in tumor tissues.

However, systemic injections of these agents pose the risk of unexpected side effects due to non-specific action—this is a major problem to be solved [15,16]. Recently, drug-delivery systems combined with micro/nanobubbles and ultrasound have been used to enhance the localization and accumulation of drugs and gene therapeutics in target tissues without damaging normal tissues [17,18,19,20]. When micro/nanobubbles are exposed to ultrasound, they alternate symmetrically, expanding and compressing with the wave’s high- and low-pressure phases. At higher ultrasound intensities, the bubbles expand rapidly and then collapse. [21,22] The physical impact (e.g., jet formation) generated at that time increases the permeability of tissues and blood vessels via perforations of membranes and vessels [23,24,25,26]. Micro/nanobubble-mediated delivery is expected to offer safe and effective antibody delivery. The size of commercial microbubbles is approximately 1–8 µm, and most of them stay in the intra-tumor vasculature because of their large size. Consequently, it is difficult for microbubble-mediated delivery to deliver drugs directly to cancer cells that are present outside of blood vessels. NBs, on the other hand, can extravasate from tumor blood vessels and penetrate deeply into tumor tissues, implying that nanobubble-mediated delivery facilitates drug delivery to tumor tissues [27,28]. We have developed a combination system with nanobubbles (NBs) and therapeutic ultrasound (TUS) that can efficiently deliver drugs and genes to target sites [29,30,31,32]. Previously, we reported that the combination of NBs and TUS exposure enhances the permeability of the blood–brain barrier (BBB), which is a strict biological barrier, and the delivery efficiency of macromolecules (e.g., pDNA and 2000 kDa dextran) [32]. In addition, in ultrasound-mediated delivery studies, drug delivery using therapeutic agent-conjugated MBs/NBs has attracted attention because of the enhanced local drug accumulation in the desired tissues [33,34]. There are several strategies for modifying antibodies onto nanocarriers, such as covalent and non-covalent binding methods [35]. We recently succeeded in the development of linker polypeptides that bind to the Fc region of IgG [36]. These polypeptides have high binding affinities because they are derived from Protein A/G, allowing for easy and rapid antibody loading [37,38]. In the current study, we developed therapeutic antibody-modified NBs using an Fc-binding linker polypeptide to establish a noninvasive and efficient antibody-delivery system with TUS exposure. We hypothesized that a combination of antibody-modified NBs using the Fc-binding linker polypeptide and TUS exposure would be useful for targeted antibody delivery into tumor tissues. In this study, we used Herceptin^®^ (trastuzumab), which targets HER2-expressing cancer cells, as a model therapeutic antibody, and quantitatively evaluated the antibody activity in therapeutic antibody-modified NBs upon exposure to TUS to assess the utility of the platform as an antibody-delivery system for cancer therapy (Figure 1).

## 2. Materials and Methods

### 2.1. Materials

Lipids, such as 1,2-distearoyl-sn-glycero-3-phosphatidylcholine (DSPC), N-(carbonyl-methoxypolyethyleneglycol2000)-1,2-distearoyl-sn-glycero-3 phosphatidyl-ethanolamine (DSPE-PEG2000), and 1,2-distearoyl-sn-glycero-3-phosphoethanolamine-N-[maleimide(polyethylene glycol)-2000] (ammonium salt) (DSPE-PEG2000-Mal), were purchased from NOF Corporation (Tokyo, Japan). Tris(2-carboxyethyl)phosphine hydrochloride (TCEP) and L-cysteine were purchased from FUJIFILM Wako Pure Chemical Corporation (Tokyo, Japan). Herceptin^®^ (trastuzumab) was purchased from Chugai Pharmaceutical Co., Ltd. (Tokyo, Japan). 1,1-dioctadecyl-3,3,3,3-tetramethyl-indocarbocyanine perchlorate (DiI) was purchased from Setareh Biotech LLC (Eugene, OR, USA).

### 2.2. Preparation of Fc-Binding Polypeptides, His-Fc-G67

His-Fc-G67 polypeptide was prepared by our previous methods with some modifications [36]. Fc-G67 contains the amino acid sequence derived from C1 region of Protein G, which strongly binds to IgG Fc region. A DNA fragment encoding the forward linker, C1 region, and the backward linker cysteine was synthesized by FASMAC Co., Ltd. (Kanagawa, Japan) and was then cloned into pET302/NT-His (Thermo Fisher Scientific, Waltham, MA, USA). The recombinant proteins were produced in BL21(DE3) cells (Thermo Fisher Scientific) using MagicMedia^TM^ E coli expression medium (Thermo Fisher Scientific) and were purified with TALON Metal Affinity Resin (Takara Bio Inc., Shiga, Japan).

### 2.3. Preparation and Characterization of Herceptin-Modified NBs (Her-NBs)

Liposomes (lipo) were prepared using the reverse-phase evaporation vesicle method, as previously described [39]. Briefly, DSPC and DSPE-PEG2000 were dissolved in chloroform/diisopropyl ether at a ratio of 1:1 (*v/v*) and then mixed at a molar ratio of 94:4. After adding phosphate-buffered saline (PBS) to the lipid solution, the mixture was sonicated at room temperature and then evaporated at 65 °C to completely remove the organic solvent. The size of the lipo was adjusted to approximately 100–200 nm using extrusion equipment by passing through sizing filters (pore sizes: 100 nm and 200 nm) and then sterilized using a 0.45 µm filter (Asahi Glass Co., Ltd., Tokyo, Japan). For fluorescent labeling of the lipid membrane, DiI (1 mol% of total lipids) was added to the lipid solution. 

Next, we modified the Fc-G67 polypeptide on the surface of the lipo using a post-insertion method [40,41]. The Fc-G67 polypeptide (0.2 mol%) was added to PBS in the presence of tris(2-carboxyethyl)phosphine chloride (TCEP, final concentration:20 mM). The dried film of DSPE-PEG2000-Mal (2 mol%) was hydrated in the PBS-containing polypeptide with gentle agitation and heating at 65 °C. After incubation at room temperature for 6 h, the Fc-G67 peptide-conjugated PEG2000-Mal-micelles were mixed with pre-formed liposomes at 60 °C for 1 h. To inactivate the free maleimide groups on the surface of the lipo, L-cysteine (final concentration: 0.1 mM) was added and incubated for 15 min at room temperature. To prepare non-modified lipo, 2 mol% of PEG2000–Mal micelles without peptide were mixed with the pre-formed lipo at 60 °C for 1 h. The resulting lipo were purified thrice by ultrafiltration (4500 rpm, 20 min, 4 °C) with 100,000 Da cutoff filter devices to remove residual TCEP and L-cysteine. Herceptin-modified lipo (Her-Lipo) were prepared by adding Herceptin to polypeptide-modified lipo at a molar ratio of 2:1 (Herceptin to Fc-G67) and incubated for 15 min at room temperature. Free-Herceptin was removed by ultracentrifugation (140,000× *g*, 20 min, 4 °C) at three times. The lipid concentration of the prepared lipo was measured using the phospholipid C-test (Wako Pure Chemical Industries Ltd., Osaka, Japan).

Each NBs was prepared as described previous report [36]. Briefly, 2 mL sterilized vials containing 0.8 mL liposome suspension (lipid concentration:1 mg/mL) were filled with perfluoropropane gas (Takachiho Chemical Inc., Co., Ltd., Tokyo, Japan), capped, and then pressurized with 3 mL of perfluoropropane gas. The vials were placed in a bath sonicator (40 kHz, Bransonic 2800-J, Branson Ultrasonics Co., Danbury, CT, USA) for 2–5 min to form NBs. The particle size of the NBs was determined via laser diffraction using a particle sizer (Aggregates Sizer, Shimadzu Co., Ltd., Kyoto, Japan). Zeta potential was measured by Laser Doppler Electrophoresis (Zetasizer Nano ZSP, Malvern Instruments, Malvern, UK).

### 2.4. Cell Cultures

SKOV3 and MDA-MB-231 cells were purchased from ATCC (Manassas, VA, USA). Jurkat cells were purchased from Promega (Madison, WI, USA). SKOV3 cells were cultured in McCoy’s 5A medium supplemented with 10% heat-inactivated fetal bovine serum (FBS), 100 U/mL penicillin, and 100 µg/mL streptomycin. MDA-MB-231 cells were cultured in Leibovitz’s L-15 Medium supplemented with 15% heat-inactivated FBS and antibiotics. Jurkat cells were cultured in RPMI1640 medium supplemented with 10% FBS, 100 U/mL penicillin, and 100 µg/mL streptomycin. SKOV3 cells and Jurkat cells were maintained at 37 °C in a humidified 5% CO_2_/95% air atmosphere. MDA-MB-231 cells were maintained at 37 °C without CO_2_.

### 2.5. Fluorescent Microscope Analysis

SKOV3 cells (0.5 × 10^4^ cells/well) were seeded on a 96-well plate the day before observation. The cells were blocked with PBS containing 1% BSA. Next, 60 µL of Her-NBs mixed with 360 µL of the medium was added to the cells. The plates were sealed and inverted for 5 min. After incubation, the plates were re-inverted for 3 min and then washed twice with PBS to remove non-attached NBs. The cells were then fixed with 4% paraformaldehyde and the nuclei were stained with DAPI. Images were captured by fluorescence microscopy (BZ-X800; KEYENCE, Osaka, Japan).

### 2.6. Evaluation of Ultrasound-Responsive Ability In Vitro

The ultrasound-responsive ability of Her-NBs in vitro was determined as follows: Ten microliters of NBs or NBs exposed to therapeutic ultrasound (TUS) were added to 5.99 mL of PBS in 6 well plate to obtain ultrasound images (B-mode) using a high-frequency ultrasound imaging system (50 MHz; NP60R-UBM, Nepa Gene, Co., Ltd., Chiba, Japan). Her-NBs were exposed to US (frequency, 1 MHz; duty, 50%; intensity, 1 W/cm^2^; time, 2 min) probe (Sonitron 2000, NEPA GENE Co., Ltd.; Chiba, Japan).

### 2.7. Analysis of ADCC Activity of Her-NBs

The antibody-dependent cellular cytotoxicity (ADCC) activity of the Her-NBs was evaluated using ADCC reporter bioassay reagents (Promega Corporation, Madison, WI, USA) [42,43]. In brief, SKOV3 cells (0.5 × 10^4^ cells/100 µL), which were the target cells, were seeded onto a 96-well plate the day before observation. The next day, after the removal of the medium, 25 µL of assay buffer (RPMI 1640 containing 4% (*v/v*) low IgG FBS) was added to each well, and samples were added. Then, Jurkat effector cells, which are effector cells expressing NFAT-RE-luc2, resuspended in assay buffer, were added to the wells at a density of 7.5 × 10^4^ cells/25 µL and incubated at 37 °C in 5% CO_2_ atmosphere. After 6 h, 75 μL of Bio-Glo^TM^ luciferase assay reagent was added to the wells and incubated at room temperature for 15 min. The resulting luminescence in the culture supernatants was measured using a Synergy HTX Multi-Mode Plate Reader (BioTek Instruments, Inc., Whiting, VT, USA).

### 2.8. Tumor Model

Female BALB/cSlc-nu/nu mice (6 weeks old) were purchased from Japan SLC Inc. (Shizuoka, Japan). SKOV3 cells (5 × 10^6^ cells) were inoculated subcutaneously into the right flank of mice. Tumor volume was calculated by measuring the length and width of the tumors with a caliper and then using the following equation: volume (V; mm^3^) = (width)^2^ × (length) × 0.5.

### 2.9. Evaluation of Ultrasound Responsive Ability in Tumor Model Mice

When the tumor reached approximately 200–300 mm^3^, Her-NBs (200 µg/200 µL) were administered to the mice under anesthesia via the tail vein in an insulin syringe with a 30-gauge needle (NIPRO Co., Ltd., Osaka, Japan), and US images of Her-NBs were observed in the tumors. US imaging was performed using an Aplio80 US diagnostic machine (Toshiba Medical Systems, Tokyo, Japan) and a 12-MHz wideband transducer with contrast harmonic imaging at a mechanical index of 0.27. In the case of TUS exposure, a TUS probe was positioned on the tumors immediately after administration and exposed to ultrasound under the same conditions as in Section 2.6.

### 2.10. Evaluation of Tumor Inhibitory Activity

When the tumor reached approximately 65 mm^3^, the mice were randomly divided into six groups (*n* = 4) as follows: PBS (control), Herceptin only, PEG-NBs + TUS, Her-NBs only, Her-NBs + TUS, and PEG-NBs + Her + TUS. Each mouse received treatment while under anesthesia via intravenous injection of NBs (200 µg/200 µL) and immediate TUS exposure at the tumor site, three times every other day. The conditions of the TUS were the same as in the above experiments. The injection dose of Herceptin was 0.4 µg per head. We measured the tumor size on days 3, 5 and 8 following the first treatment day (day 1), and then calculated the relative tumor volume (V/V_0_; V_0_ is tumor volume on day 1). Body weight was measured in the same manner.

### 2.11. Statistical Analysis

The mean SD (*n* = 3 or 4) was used to represent all data. The results were considered significant when *p* < 0.05. To determine statistical significance, the unpaired *t*-test (two groups) or one-way ANOVA followed by the Tukey test (more than three groups) was performed.

## 3. Results

### 3.1. Characterization of Her-NBs

**Figure 2:**
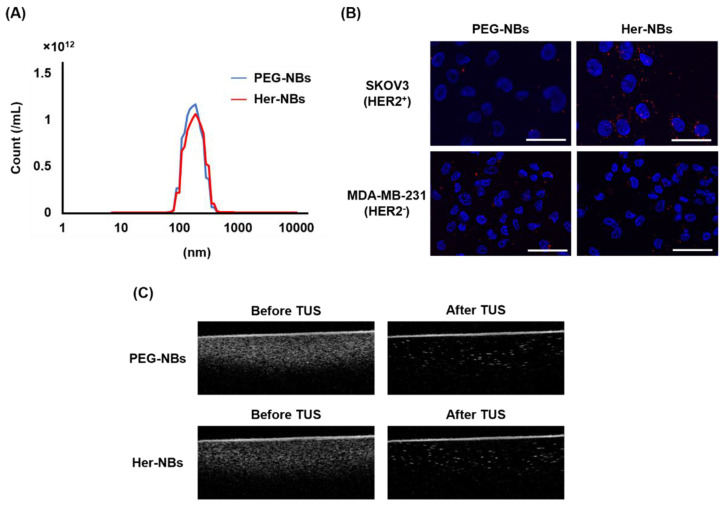
Characterization of non-modified PEG-NBs and Her-NBs. (**A**) Particle size distribution was measured via Laser Diffraction Method. (**B**) Attachment of Her-NBs and cancer cells. DiI-labeled NBs were incubated with SKOV3 (HER2^+^ cancer) cells or MDA-MB-231 (HER2^−^ cancer) cells for 5 min, and then DAPI was added to the cells to stain nuclei. Red: DiI-labeled NBs, Blue: DAPI, scale bar: 50 µm. (**C**) Ultrasound contrast images of NBs before/after therapeutic ultrasound (TUS) exposure (frequency, 1 MHz; duty, 50%; intensity, 1 W/cm^2^; time, 2 min).

We first evaluated the average sizes and zeta-potentials of the non-modified PEG-NBs and Her-NBs. Both PEG-NBs and Her-NBs were approximately 170 nm in size (Table 1). The size distributions of NBs were relatively narrow, and no significant difference between PEG-NBs and Her-NBs was observed (Figure 2A). The zeta potential of both NBs was approximately −20 mV. To evaluate the modification of Herceptin in Her-NBs, the attachment of DiI-labeled Her-NBs to HER2 positive (SKOV3) cells or negative (MDA-MB-231) cells was observed by fluorescence microscopy. As shown in Figure 2B, in SKOV3 cells, higher fluorescence was observed in the Her-NB treatment group than in the PEG-NB treatment group. In MDA-MB-231 cells, fluorescence was rarely observed in either group of cells. These results suggest that Herceptin is retained on the surface of the NBs. 

Next, we confirmed the ultrasound responsiveness of Her-NBs. Ultrasound images of NBs before and after therapeutic US (TUS) exposure are shown in Figure 2C. The ultrasound echo signals decreased after exposure to TUS. This result suggests that exposure to TUS (1 MHz, 1 W, 2 min, duty cycle 50%) induces cavitation and collapse of the NBs.

### 3.2. Antibody Activity of Herceptin on Her-NBs

**Figure 3:**
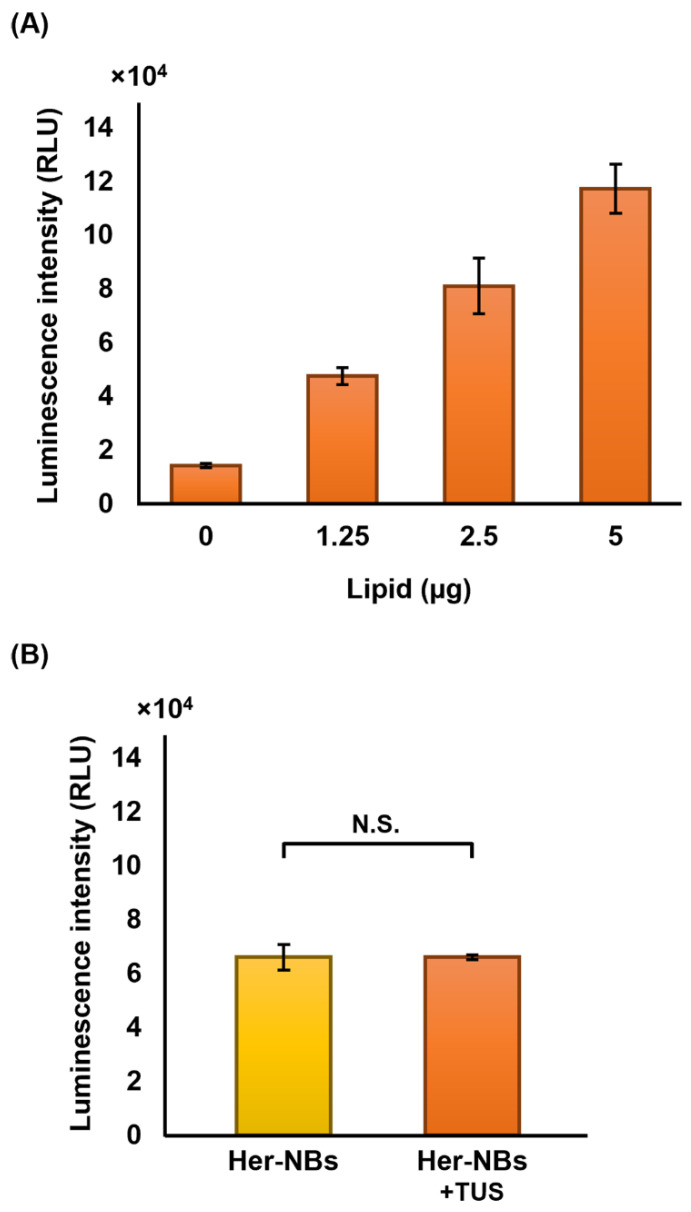

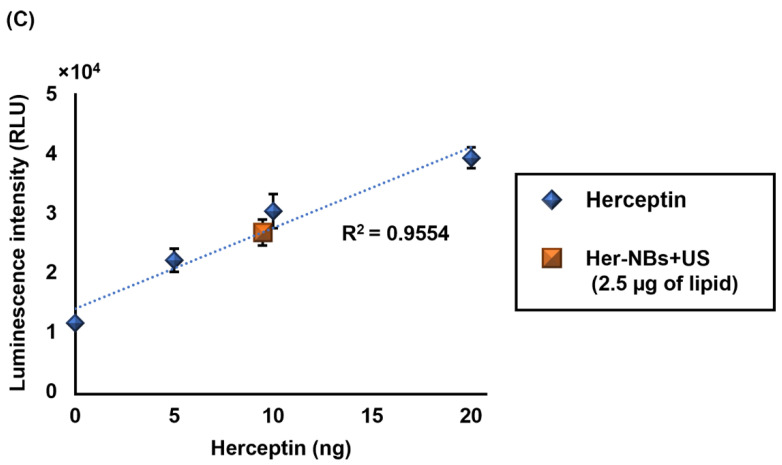
Activity of Herceptin on Her-NBs before/after TUS exposure. To evaluate the activity of Herceptin, luminescence intensity of Jurkat effector cells, expressing NFAT-RE-luc2, was measured by ADCC reporter bioassay against SKOV3 (T). SKOV3 cells were plated in a 96-well plate the before assay. On the day of assay, samples and effector cells (E) were added to the plate (cell rate; E:T = 15:1). After 6 h of incubation at 37 °C/5% CO_2_ condition, Bio-Glo^TM^ luciferase assay regent was added, and the luminescence intensity was determined using luminometer. (**A**) Luminescence intensity of Herceptin in Her-NBs after TUS in serial concentration of lipid. (**B**) Comparison of luminescence intensity of Her-NBs before and after TUS exposure in 2.5 µg of lipid. (**C**) Calculation of titer of Herceptin in Her-NBs using the standard curve of the amount of Herceptin versus luminescence intensity. Data = mean ± SD (*n* = 4). The unpaired *t*-test was used for statistical analysis.

To clarify the effects of ultrasound exposure, the activity of Herceptin on Her-NBs after TUS exposure was evaluated by quantifying NFAT-mediated luciferase activity in effector cells using the ADCC reporter bioassay. As shown in Figure 3A, the luminescence intensity of the treatment group of Her-NBs after TUS exposure increased with increasing lipid concentration, whereas that of the treatment group of PEG-NBs after TUS exposure was similar to that of the control group (Figure S1). These results suggest that the activity of Herceptin remained after TUS exposure. In addition, when the luminescence intensities of Her-NBs after TUS exposure were compared with those of Her-NBs before TUS exposure, these values were almost identical (Figure 3B). This suggests that TUS exposure did not affect the antibody activity. To measure the amount of Herceptin on Her-NBs, the antibody titer of Herceptin in Her-NBs was calculated using a calibration curve (R2 value ≥ 0.95) of the luminescence intensity of Herceptin alone (Figure 3C). We found that the titer was approximately 0.38 ± 0.06 µg of Herceptin per 100 µg of lipid in Her-NBs.

### 3.3. Evaluation of Responsive of Her-NBs to Therapeutic Ultrasound In Vivo

**Figure 4:**
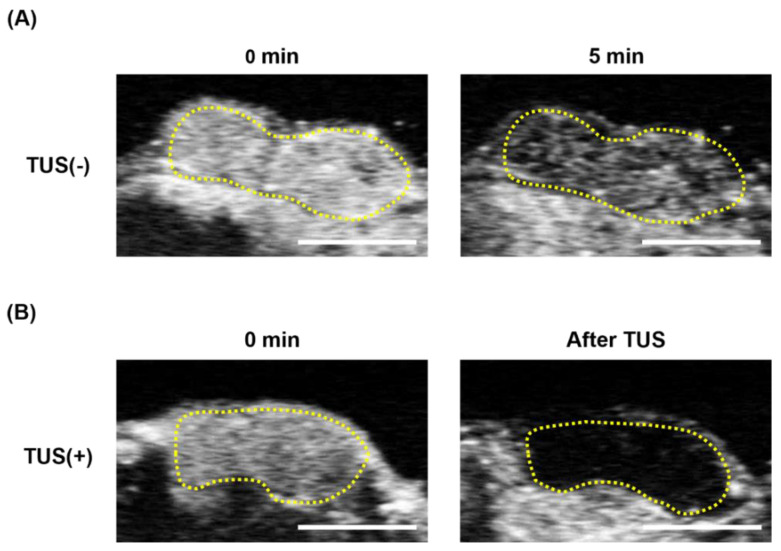
Ultrasonography images of Her-NBs with/without TUS groups in tumors in vivo. SKOV3-bearing mice (Tumor volume: approximately 200–300 mm^3^) were injected with Her-NBs intravenously, and contrast images of the tumors were captured. The tumor was exposed to TUS (frequency, 1 MHz; duty, 50%; intensity, 1 W/cm^2^; time, 2 min) immediately after injection in the TUS-exposure groups. Contrast images of (**A**) Her-NBs without TUS from 0 min to 5 min and (**B**) Her-NBs with TUS at 0 min and immediately after TUS exposure. Images were captured by US diagnostic machine with a 12 MHz probe. The circles show the area of the tumor. Scale bar: 5 mm.

To confirm the cavitation behavior of Her-NBs induced by ultrasound exposure in vivo, we performed US imaging in tumor-bearing mice. Ultrasound echo signals were observed clearly after 5 min the administration of Her-NBs via the tail vein (Figure 4A). In contrast, no echo signals were observed immediately after the TUS exposure (Figure 4B). These results demonstrate that cavitation accompanied by the collapse of NBs also occurred in vivo after ultrasound exposure, similar to that observed in vitro.

### 3.4. Anti-Tumor Effect of the Combination Treatment of Her-NBs and TUS Exposure

**Figure 5:**
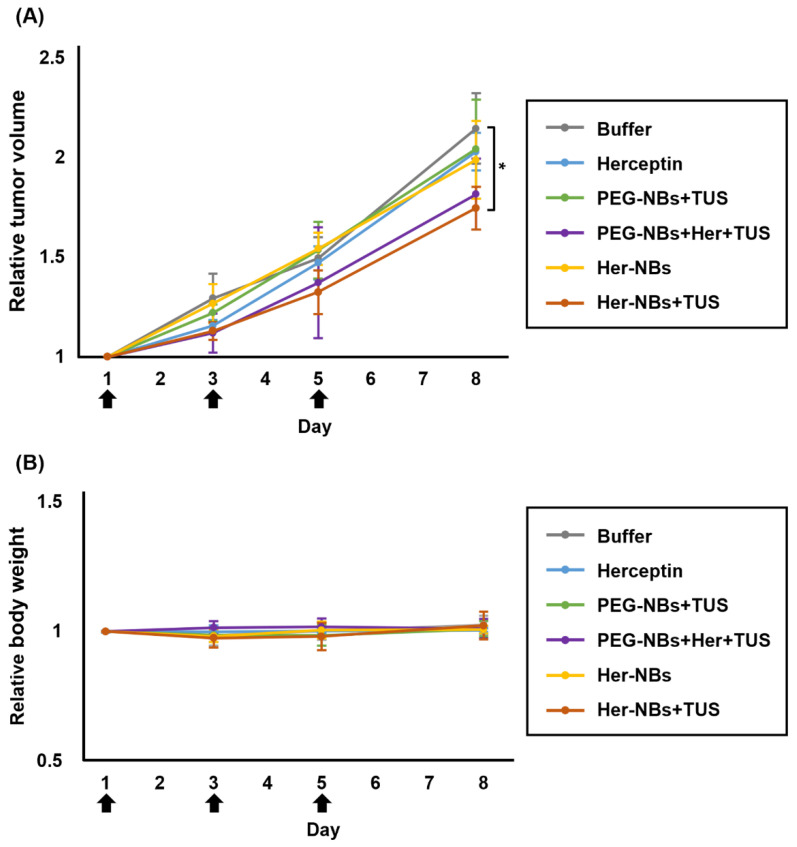
In vivo efficacy of the combination of Her-NBs and TUS exposure against SKOV3 tumor models. Tumor growth rate (**A**) and changes in body weight (**B**) on day 8. Tumor-bearing mice (tumor volume: approximately 65 mm^3^) were injected with PBS, Herceptin (0.8 μg/head), or NBs three times every other day. The black arrows indicate treatment days. The tumor was exposed to TUS (frequency, 1 MHz; duty, 50%; intensity, 1 W/cm^2^; time, 2 min) immediately after injection in the TUS-exposure groups. Relative tumor volumes and relative body weights are expressed as multiples of the initial volumes on day 1. Data = mean ± SD (*n* = 4). * *p* < 0.05 (one-way ANOVA followed by the Tukey’s test).

To confirm whether the combination of antibody-modified NBs and TUS exposure is useful for delivery systems in cancer treatment, the therapeutic effects of the combination of Her-NBs and TUS exposure were examined in vivo in mice with SKOV3 tumors. All the mice were treated three times every other day and exhibited no apparent signs of distress. Tumor growth rates in SKOV3 xenografted mice were calculated as one on the day of treatment initiation, and the growth rates on 1, 3, 5 and 8 in Figure 5. As shown in Figure 5A, the tumor growth rate on day 8 in the treatment group of Her-NBs and TUS exposure was the lowest among the other groups. None of the animals lost weight significantly or died during the experiment (Figure 5B). This result suggests that the combination of antibody-modified NBs and TUS exposure can efficiently deliver therapeutic antibodies to target tumors and enhance the therapeutic effect of antibodies.

## 4. Discussion

The efficacy and tumor accumulation of therapeutic antibodies go hand-in-hand with successful solid cancer therapy. However, the penetration of therapeutic antibodies into tumors is limited by the tumor microenvironment (TME), resulting in reduced therapeutic efficacy and drug resistance [8]. Therefore, the development of antibody-delivery systems is required to overcome these barriers to enhance antibody permeability. Combination therapy with agents such as hyaluronidase may mitigate tumor barriers. However, the combination therapy has not been used widely for clinical applications since multiple organ damages were caused by these agents [15,16]. Therefore, the development of noninvasive approaches for antibody delivery is essential for use in clinical settings. Here, we focused on micro/nanobubble-mediated drug delivery systems. The combination of micro/nanobubbles and therapeutic ultrasound (TUS) has been suggested as a safe and effective method for delivering drugs to the target organs [17,18,19,20]. 

In this study, to achieve more efficient antibody delivery, we developed therapeutic antibody-modified nanobubbles (NBs) using Fc-G67 polypeptides, which bind strongly to the Fc region of the antibodies. To evaluate the utility of our combination of antibody-modified NBs and TUS as antibody delivery systems for tumors, we examined the antibody activity of antibody-modified NBs and the anticancer therapeutic effect of the combination of antibody-modified NBs and TUS exposure. First, we developed Herceptin-modified nanobubbles (Her-NBs) as antibody delivery carriers using Fc-binding polypeptides. We achieved antibody modification of the NBs by mixing for 15 min at room temperature. The Her-NBs have monodisperse particles with an average size of approximately 170 nm (Table 1 and Figure 2). Because the pore size of normal blood vessels is approximately 7 nm, the particle size cannot leak from blood vessels into healthy tissues. In tumor tissue, however, because the pore size of tumor blood vessels is approximately 0.4–2 µm, the particle size can extravasate through the tumor vasculature [44,45]. Therefore, Her-NBs can permeate deep tissues in tumors without penetrating healthy tissues. Next, antibody modification was qualitatively evaluated by the attachment of Her-NBs and cancer cells (Figure 2B). We observed specific attachment between Her-NBs and HER2-positive cancer cells and confirmed Herceptin modification on Her-NBs. These results suggest that the method using Fc-binding polypeptides enables the rapid development of therapeutic antibody-modified nanocarriers in a short time. Cavitation induced by nanobubbles was examined using in vitro ultrasound imaging. As shown in Figure 2C, it was confirmed that cavitation was induced with or without antibody modification. 

Although delivery strategies using Fc region-binding proteins have been studied, few studies have quantitatively evaluated the biological activity of these antibodies. Therefore, we evaluated the activity of Herceptin on Her-NBs using our combination strategy. Various factors (e.g., Fc-binding polypeptide and ultrasound exposure) may affect the activity of antibodies in our combination system. Antibody-dependent cellular cytotoxicity (ADCC) is one of the critical mechanisms of action of antibodies and is thought to occur when immune cells with Fc receptors recognize the CH2 domain of the Fc region of antibodies bound to target tumor cells [46]. Fc-binding polypeptides generally bind to the CH2-CH3 domain of the Fc region [47]. Therefore, immune cells cannot recognize the CH2 domain due to steric hindrance, which may reduce ADCC activity. In addition, mechanical and thermal effects generated by ultrasound exposure and nanobubble cavitation may induce structural changes in the antibodies. To evaluate the effect of the Fc-G67 polypeptide and ultrasound exposure, we used an ADCC reporter bioassay (Figure 3). The ADCC activity of Her-NBs did not change, even after TUS exposure. This result demonstrates that the ultrasound exposure conditions applied here do not affect the structure of the antibody or the binding affinity. Moreover, the amount of Herceptin on Her-NBs was calculated from ADCC reporter signals to be approximately 0.4 µg of Herceptin per 100 µg lipid. Previously, the amount of Herceptin on Her-Lipo was evaluated semi-quantitatively by using SDS-PAGE and silver staining—Her-Lipo (per 100 µg lipid) has approximately 0.4 µg of Herceptin (no data shown). Both were very similar. These results imply that Fc-binding polypeptides do not decrease the ADCC activity. Because immune cells either avoid the binding site of the Fc-binding polypeptide or bind to another side where the peptide does not bind, we determined that immune cells (i.e., Jurkat effector cells) can recognize the CH2 domain of the antibody. These results suggest that antibody-modified NBs using Fc-binding polypeptides are useful for ultrasound antibody delivery carriers that do not inactivate antibodies. 

Our laboratory previously reported that the combination of NBs and TUS exposure could enhance blood vessel permeability and improve the accumulation of micromolecular agents [32]. In this study, we attempted antibody delivery to tumor tissues by the combination of Her-NBs and TUS exposure and evaluated the therapeutic effect in SKOV3 tumor-bearing mice. As shown in Figure 5A, the Her-NBs + TUS group exhibited a higher tumor-inhibitory effect than the other groups. In contrast, the Her-NBs without TUS group exhibited no significant tumor inhibition. The tumor inhibitory effect of the Her-NBs + TUS group may be due to the accumulation of Herceptin because Her-NBs and TUS exposure enhanced the permeability of tumor tissue. Another factor may be the direct damage to the tumor tissue caused by the mechanical and thermal effects of cavitation. However, we believe that this possibility is extremely low because there was no difference between the inhibitory effect of the PEG-NBs + TUS group and the control group. Moreover, to confirm whether antibody modification of NBs enhances the efficiency of antibody delivery, we compared the non-modified PEG-NBs + Her + TUS group with the Her-NBs + TUS group. There was not significant difference. The low inhibitory effect of Herceptin alone may be due to its low dosage. The dose of Herceptin in this study was significantly lower than that in other studies because of the adjustment to the dosage of Her-NBs [48,49]. Thus, an increased modification of Herceptin to Her-NB would improve the therapeutic effect of the combination of Her-NB and TUS. No obvious weight loss was confirmed in the Her-NBs + TUS group, suggesting that this combination therapy is a safe delivery system. Taken together, these results suggest that the combination of Her-NBs and TUS may be an efficient system for maximizing the therapeutic potential of antibody drugs in cancer tissues.

Although the combination of Her-NBs and TUS was useful for antibody delivery, there were some key limitations. First, the loading amount of antibodies in the NBs was low. The tumor inhibitory effect in the Her-NBs + TUS group was higher than that in the Herceptin group, although the difference was not statistically significant. This finding may be attributed to the low Herceptin injection dose, since tumor inhibitory effects were almost the same between the PBS and Herceptin groups (injected dose was adjusted to the Herceptin loading amount in Her-NBs). In the future, we plan to explore methods for improving the loading amount of therapeutic antibodies (e.g., by optimizing the modification of linker polypeptides, exploring new polypeptide-modification methods, and changing other peptides with short amino acid sequences). Second, antibody behavior after TUS exposure remains unclear. It remains to be determined how much antibody can be delivered to the tumor and where antibodies are located in the tumor. We need to perform histochemical analysis to elucidate the mechanism of antibody delivery. Third, as this study used a mouse model of subcutaneous cancer xenografts, which has a low volume of tumor stroma [50,51], it is necessary to validate the results in a cancer stroma-rich model. This was only a proof-of-concept study of a combination system using Her-NBs and TUS for antibody delivery to tumors. However, to consider clinical application, it is necessary to evaluate the antibody delivery ability of the system in tumor-tissue models (such as organoids), which are more similar to human cancer tissues. When the stroma is abundant in the tumor, it may be difficult to apply the same ultrasound exposure conditions; therefore, conditions may need to be optimized, or focused ultrasound (FUS) may need to be used.

## Figures and Tables

**Table 1:** Average particle size and zeta potential of PEG-NBs and Her-NBs (*n* = 3).

Nanobubbles	Size (nm)	Zeta Potential (mV)
PEG-NBs	165.7 ± 4.6	−24.0 ± 2.6
Her-NBs	172.3 ± 1.5	−21.6 ± 1.1

## Data Availability

The data presented in this study are available in the article and supplementary material.

## Supplementary Materials

The following supporting information can be downloaded at: https://www.mdpi.com/article/10.3390/pharmaceutics15010130/s1, Figure S1: ADCC activity of PEG-NBs after TUS exposure (5 µg of lipid). Data = mean ± SD (*n* = 4).
