# Supplementary material for: Development of an Antibody Delivery Method for Cancer Treatment by Combining Ultrasound with Therapeutic Antibody-Modified Nanobubbles Using Fc-Binding Polypeptide
Source: Pharmaceutics. 2022 Dec 30;15(1):130. doi: 10.3390/pharmaceutics15010130 (PMC9861716; doi:10.3390/pharmaceutics15010130)
Supplement: Supplementary file 1 [file pharmaceutics-15-00130-s001.zip › pharmaceutics-2096574-supplementary.pdf]

Supplementary Materials

Development of an Antibody Delivery Method for Cancer Treatment by Combining Ultrasound with Therapeutic Antibody-Modified Nanobubbles Using Fc-Binding Polypeptide

Yusuke Yano [1,†], Nobuhito Hamano [1,†], Kenshin Haruta [1], Tomomi Kobayashi [1], Masahiro Sato [1], Yamato Kikkawa [2], Yoko Endo-Takahashi [1], Rui Tada [1], Ryo Suzuki [3], Kazuo Maruyama [4], Motoyoshi Nomizu [2] and Yoichi Negishi [1,*]

[1] Department of Drug Delivery and Molecular Biopharmaceutics, School of Pharmacy, Tokyo University of Pharmacy and Life Sciences, 1432-1 Horinouchi, Hachioji, Tokyo 192-0392, Japan
[2] Department of Clinical Biochemistry, School of Pharmacy, Tokyo University of Pharmacy and Life Sciences, Tokyo 192-0392, Japan
[3] Laboratory of Drug and Gene Delivery Research, Faculty of Pharma-Sciences, Teikyo University, 2-11-1 Kaga, Itabashi-ku, Tokyo 173-8605, Japan
[4] Laboratory of Ultrasound Theranostics, Faculty of Pharma-Sciences, Teikyo University, 2-11-1 Kaga,
Itabashi-ku, Tokyo 173-8605, Japan
* Correspondence: negishi@toyaku.ac.jp; Tel.: +81-42-676-3183
† These authors contributed equally to this work.

Figure. S1 Experimental section

ADCC activity of PEG-NBs after TUS exposure

The preparation of PEG-NBs is same as the manuscript. SKOV3 (0.5×10^4 cells/ 100 μL /well) were seeded onto a 96-well plate the day before observation and incubated overnight at 37°C in 5% CO_2. The next day, after the removal of the medium, 25 μL of assay buffer (RPMI 1640 containing 4% (v/v) low IgG FBS) was added to each well, and PEG-NBs (5 μg as a Lipid) which was exposed to TUS were added. Then, Jurkat effector cells were added to the wells at a density of 7.5×10^4 cells/ 25 μL and incubated at 37℃ in 5% CO_2 atmosphere. After 6 h, 75 μL of Bio-Glo^{TM} luciferase assay reagent was added to the wells and incubated at room temperature for 15 min. The resulting luminescence in the culture supernatants was measured using a Synergy HTX Multi-Mode Plate Reader (BioTek Instruments, Inc., Whiting, VT, USA). TUS condition is same as the manuscript.

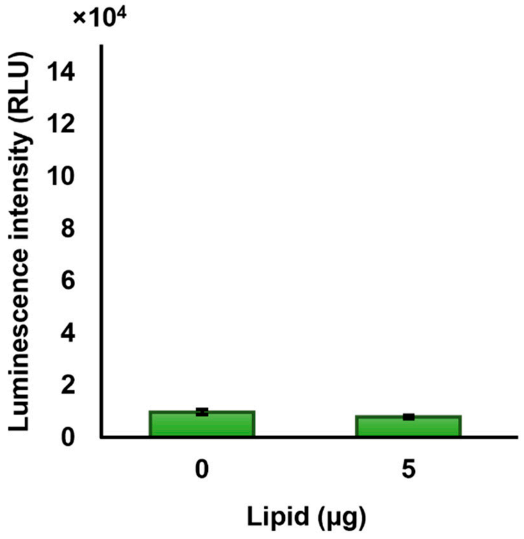

Supplement Figure S1

Luminescence intensity of PEG-NBs exposed ultrasound

To evaluate the ADCC activity, luminescence intensity of Jurkat effector cells, expressing NFAT-RE-luc2, was measured by ADCC reporter bioassay against SKOV3 (T). SKOV3 cells were plated in a 96-well plate the before assay. On the day of assay, the samples and effector cells (E) were added to the plate (cell rate; E: T= 15: 1). After 6 h of incubation at 37℃/5% CO_2 condition, Bio-Glo™ luciferase assay regent was added, and the luminescence intensity was determined using luminometer.
